# Preventing continuous renal replacement therapies (CRRT)-induced hypophosphatemia using a phosphate-containing CRRT solution in the setting of regional citrate anticoagulation

**DOI:** 10.1186/cc13583

**Published:** 2014-03-17

**Authors:** V Pistolesi, S Morabito, L Tritapepe, E Vitaliano, L Zeppilli, F Polistena, MI Sacco, E Fiaccadori, A Pierucci

**Affiliations:** 1 Umberto I, Policlinico di Roma, Rome, Italy; 2 Pertini Hospital, Rome, Italy; 3 University of Parma Medical School, Parma, Italy

## Introduction

Phosphate depletion is a known issue during continuous renal replacement therapies (CRRT) with an incidence of hypophosphatemia up to 80% when standard CRRT solutions are used. The aim was to evaluate the effects on serum phosphate and phosphorus supplementation needs of a regional citrate anticoagulation (RCA) protocol for CRRT combining the use of citrate with a phosphate-containing CRRT solution.

## Methods

In critically ill heart surgery patients undergoing CRRT for acute kidney injury, we adopted RCA in a CVVH or CVVHDF modality combining a commercially available citrate solution (18 mmol/l) with a phosphate-containing CRRT solution as dialysate and/or replacement fluid (HCO_3 _- 30 mmol/l, phosphate 1.2). The prescribed CRRT dose, corrected for predilution, was at least 25 ml/kg/hour with about 50 to 60% of dialysis dose given as phosphate-containing solution. By convention, hypophosphatemia was defined as follows: mild (<0.81 mmol/l), moderate (<0.61 mmol/l) and severe (<0.32 mmol/l).

## Results

Forty-eight patients were treated with RCA-CRRT for at least 72 hours (total running time 12,502 hours). Two-hundred and nineteen RCA-CVVH circuits were used with a mean filter life of 57.1 ± 41.7 hours (median 47, IQR 24 to 83). Acid-base status was adequately maintained without the need for additional interventions on RCA-CRRT parameters (pH 7.43 (7.40 to 7.47), bicarbonate 25.3 mmol/l (23.8 to 26.6), BE 0.9 (−0.7 to 2.4); median (IQR)). Serum phosphate was steadily maintained in a narrow range throughout RCA-CRRT days (1.2 mmol/l (0.97 to 1.45); median (IQR)). At some times during CRRT, only 10 out of 48 patients (20.8%) received a low amount of phosphate supplementation (D-fructose-1,6-diphosphate 1.05 ± 2.04 g/day) for mild (*n *= 7) to moderate *(n *= 3) hypophosphatemia. In particular, considering all patients, only 33 out of 513 serum phosphorus determinations met the criteria for mild (*n *= 24) to moderate (*n *= 9) hypophosphatemia. Severe hypophosphatemia was never observed.

## Conclusion

The use of a phosphate-containing CRRT solution, accounting for about 50 to 60% of the CRRT dose in the setting of RCA-CVVH or RCA-CVVHDF, allowed one to prevent CRRT-induced phosphate depletion in most of the patients, minimizing the need for phosphate supplementation and maintaining phosphorus levels in a near-normal range throughout CRRT days.

